# Hyponatremia and the risk of Osmotic Demyelination Syndrome: is it time to review sodium correction rates?

**DOI:** 10.1590/2175-8239-JBN-2026-E005en

**Published:** 2026-01-23

**Authors:** Carlos Perez Gomes

**Affiliations:** 1 Universidade Federal do Rio de Janeiro, Rio de Janeiro, RJ, Brazil.

Severe hyponatremia, defined as natremia <120 mmol/L regardless of neurological symptoms or signs, or natremia between 120 and 129 mmol/L accompanied by severe neurological manifestations of encephalic edema (such as seizures, coma, respiratory depression, etc.), is a frequent electrolyte emergency in clinical practice. Management involves a critical balance between increasing natremia to prevent worsening encephalic edema and death, while at the same time avoiding excessively rapid correction, which has historically been associated with the dreaded osmotic demyelination syndrome (ODS). The most recent recommendations from European and American societies set conservative limits for increasing natremia (typically ~6–8 mmol/L in 24 h), precisely to prevent the development of ODS^1,2^. Paradoxically, large observational studies and meta-analyses in recent years indicate that the risk of ODS is rare and not always related to overcorrection, and that inadvertently faster correction is itself associated with lower in-hospital mortality and shorter length of stay^3,4^.

In a very well-designed observational study by Reis *et al*.^5^ published in this issue of the Brazilian Journal of Nephrology (“Overcorrection of severe hyponatremia, osmotic demyelination syndrome and mortality: insights from two Brazilian centers”), the authors retrospectively evaluated 362 patients with initial natremia <120 mmol/L admitted to two Brazilian tertiary hospitals. Overcorrection was defined as an increase in serum sodium levels of >8 mmol/L in 24 hours or >18 mmol/L in 48 hours. In addition to analyzing the time course of serum sodium correction and the potential outcomes of ODS — death and length of hospital stay — several variables (epidemiological data, comorbidities, concomitant medications, laboratory tests, specific treatment with isotonic or hypertonic saline, water restriction, potassium replacement, and/or use of furosemide) were evaluated using multiple regression and propensity score methods to identify independent predictors of overcorrection of hyponatremia and mortality.

Regarding the main findings, the mean serum sodium on admission was 113.3 ± 4.9 mmol/L, and 81.8% of patients were admitted to the intensive care unit. A total of 73.2% received 0.9% NaCl, and only six patients were treated with 3% NaCl. Overcorrection occurred in 38.7% of patients, with a diagnosis of ODS in only one patient (0.28%) and an overall mortality rate of 26.2%. The authors identified younger age, lower natremia on admission, and higher volumes of 0.9% NaCl administered in the emergency room as independent factors associated with the risk of overcorrection, while a diagnosis of cancer and the use of furosemide were protective factors.

The main causes of death were sepsis (55.8%) and cancer (15.8%), whereas aspiration of gastric contents (13.7%) was considered a possible hyponatremia-related cause of death. Five out of a total of 95 deaths were considered potentially associated with severe hyponatremia, four of which occurred in the group without overcorrection and one in the group with overcorrection. The overcorrection group had a 45% reduction in the chances of mortality (OR 0.548; 95% CI 0.389–0.772; p < 0.001). In line with the literature, sodium overcorrection was associated with lower in-hospital mortality and shorter hospital stays. However, after a detailed review of the causes of death, the authors themselves found no direct causal relationship between the sodium correction rate and mortality.

Why are these results contradictory? It is essential to distinguish observational association from causality. Some critical points, discussed even in this study, deserve consideration: 1) Indication bias and type of treatment: Patients who receive faster correction may be those with a clinical presentation that allows for more aggressive interventions (acute hyponatremia, reversible causes, closer vigilance, availability of intensive care), while patients with chronic hyponatremia, those who are very frail, or those with comorbidities receive slower corrections as a precaution. Thus, the lower mortality associated with faster correction may reflect baseline differences between groups rather than a direct effect of the speed of treatment^6^. 2) Biases in data collection and the etiology of hyponatremia: In retrospective studies, the frequency of natremia measurements and the control of variables (urinary osmolality, urine output, potassium replacement, etc.) vary greatly, which hinders the precise attribution of “overcorrection” to the therapy administered alone. Another determining factor is the different causes of hyponatremia, which also interfere with the speed of correction. For example, in hypovolemic patients, after hemodynamic stabilization, there may be a sudden increase in water diuresis due to the cessation of ADH action, promoting unintentional rapid correction, unlike euvolemic patients with SIADH, in whom the ADH stimulus is constant. 3) Low absolute risk of ODS, but high impact on the patient’s health: Although ODS is rare, when it occurs it is characterized by high morbidity and mortality. Therefore, even a small increase in risk can be considered clinically relevant^6,7^. This is at the core of the conservatism of current guidelines.

Although observational studies indicate that faster correction can reduce complications of hyponatremia, such as length of stay and even mortality, there is no causal evidence that this acceleration improves prognosis, due to the lack of randomized clinical trials. The risk of ODS, although rare, is catastrophic, which for the time being justifies the individualized approach recommended by current guidelines. These guidelines even emphasize frequent monitoring and “reversal” measures (e.g., reinfusion of hypotonic solution and/or desmopressin) in the event of unintentional overcorrection, especially in patients at higher risk of ODS (malnutrition, alcoholism, severe hypokalemia, chronic liver disease)^2,6,7^.

In summary, this original study reproduces the outcomes of other international studies in a Brazilian population, making a significant contribution to the heated discussion on the complications of treating severe hyponatremia, including the possibility of future adjustments to the guidelines themselves.

## Data Availability

No new data were generated or analyzed in this study.
