# Supplementary material for: Hyponatremia and the risk of Osmotic Demyelination Syndrome: is it time to review sodium correction rates?
Source: J Bras Nefrol. 2026 Jan 23;48(1):e2026E005. doi: 10.1590/2175-8239-JBN-2026-E005en (PMC12844543; doi:10.1590/2175-8239-JBN-2026-E005en)
Supplement: Translation

# Translation: Hiponatremia e o risco da Síndrome de Desmielinização Osmótica: é hora de rever os limites de correção do sódio?

**DOI:** 10.1590/2175-8239-JBN-2026-E005pt

**Authors:** Carlos Perez Gomes

**Affiliations:** 1 Universidade Federal do Rio de Janeiro, Rio de Janeiro, RJ, Brasil.

A hiponatremia grave, definida por natremia < 120 mmol/L, independentemente de sintomas ou sinais neurológicos, ou por natremia entre 120 e 129 mmol/L, acompanhada de sintomas neurológicos graves decorrentes de edema encefálico (tais como convulsões, coma, depressão respiratória etc.), é uma emergência eletrolítica frequente na prática clínica. O tratamento envolve um equilíbrio crítico entre aumentar a natremia para evitar piora do edema encefálico e óbito e, ao mesmo tempo, evitar correção excessivamente rápida, historicamente associada à temível síndrome de desmielinização osmótica (SDO). As recomendações mais recentes de sociedades europeias e americanas estabelecem limites conservadores para o aumento da natremia (tipicamente ~6–8 mmol/L em 24 h), justamente para prevenir o desenvolvimento da SDO^1,2^. Paradoxalmente, grandes estudos observacionais e meta-análises publicados nos últimos anos indicam que o risco de SDO é raro e nem sempre está relacionado à hipercorreção, além de que a própria correção inadvertidamente mais rápida estaria associada a menor mortalidade intra-hospitalar e menor tempo de internação^3,4^.

Em um estudo observacional muito bem delineado por Reis et al.^5^ e publicado nesta edição do *Brazilian Journal of Nephrology* (“Hipercorreção da hiponatremia grave, síndrome de desmielinização osmótica e mortalidade: percepções de dois centros brasileiros”), os autores avaliaram retrospectivamente 362 pacientes com natremia inicial < 120 mmol/L internados em dois hospitais terciários brasileiros. A hipercorreção foi definida por aumento do sódio sérico > 8 mmol/L em 24 horas ou > 18 mmol/L em 48 horas. Além da análise da evolução temporal da correção do sódio sérico e dos possíveis desfechos de SDO — óbito e tempo de internação hospitalar — diversas variáveis (dados epidemiológicos, comorbidades, medicações concomitantes, exames laboratoriais, tratamento específico com salina isotônica ou hipertônica, restrição hídrica, reposição de potássio e/ou uso de furosemida) foram analisadas por regressão múltipla e *propensity score* para identificar preditores independentes de hipercorreção da hiponatremia e de mortalidade.

Em relação aos principais achados, o sódio sérico médio na admissão foi de 113,3 ± 4,9 mmol/L, e 81,8% dos pacientes foram admitidos em unidade de terapia intensiva. Do total, 73,2% receberam NaCl 0,9% e apenas seis pacientes foram tratados com NaCl 3%. Houve hipercorreção em 38,7% dos pacientes, com diagnóstico de SDO em apenas um caso (0,28%) e mortalidade geral de 26,2%. Os autores identificaram menor idade, níveis mais baixos de natremia na admissão e maiores volumes de NaCl 0,9% administrados na emergência como fatores independentes para risco de hipercorreção, enquanto o diagnóstico de câncer e o uso de furosemida foram fatores protetores.

As principais causas de óbito foram sepse (55,8%) e câncer (15,8%), sendo a aspiração de conteúdo gástrico (13,7%) considerada uma possível causa de óbito por hiponatremia. Cinco de um total de 95 óbitos foram considerados potencialmente associados à hiponatremia grave, quatro dos quais ocorreram no grupo sem hipercorreção e um no grupo com hipercorreção. O grupo de hipercorreção da hiponatremia apresentou redução de 45% nas chances de mortalidade (OR 0,548; IC 95% 0,389 a 0,772; p < 0,001). Em consonância com a literatura, a hipercorreção do sódio foi associada a menor mortalidade intra-hospitalar e a internações mais curtas. Porém, após revisão detalhada das causas de mortalidade, os próprios autores não encontraram relação causal direta entre taxa de correção de sódio e óbito.

Por que esses resultados são contraditórios? É fundamental distinguir associação observacional de causalidade. Alguns pontos críticos, discutidos inclusive neste estudo, merecem consideração: 1) Vieses de indicação e tipo de tratamento: Pacientes que recebem correção mais rápida podem ser aqueles com apresentação clínica que permite intervenções mais agressivas (hiponatremia aguda, causas reversíveis, maior vigilância, disponibilidade de terapia intensiva), enquanto pacientes com hiponatremia crônica, muito frágeis ou com comorbidades recebem correções mais lentas por precaução. Assim, a menor mortalidade associada à correção mais rápida pode refletir diferenças basais entre os grupos, e não um efeito direto da velocidade do tratamento^6^. 2) Vieses de coleta de dados e etiologia da hiponatremia: Em estudos retrospectivos, a frequência de medição da natremia e o controle de variáveis (osmolalidade urinária, diurese, reposição de potássio etc.) variam muito, o que prejudica a atribuição precisa de “correção excessiva” somente à terapia administrada. Outro fator determinante são as diferentes causas de hiponatremia, que também interferem na velocidade de correção. Por exemplo, em pacientes hipovolêmicos, após a estabilização hemodinâmica, pode ocorrer aumento súbito da diurese aquosa pela cessação da ação do ADH, promovendo correção rápida não intencional, diferentemente do que ocorre em pacientes euvolêmicos com SIAD, cujo estímulo de ADH é constante. 3) Risco absoluto baixo de SDO, mas de alto impacto na saúde do paciente: Embora a SDO seja rara, quando ocorre é caracterizada por grande morbimortalidade. Portanto, mesmo um pequeno aumento de risco pode ser considerado clinicamente relevante^6,7^. Esse é o cerne do conservadorismo das atuais g*uidelines*.

Apesar de estudos observacionais indicarem que a correção mais rápida pode reduzir complicações da hiponatremia, como tempo de internação e até mesmo mortalidade, não há evidência causal de que essa aceleração melhore o prognóstico, devido à falta de ensaios clínicos randomizados. O risco de SDO, embora raro, é catastrófico, justificando, por ora, a abordagem individualizada preconizada pelas diretrizes atuais, que enfatizam, inclusive, monitorização frequente e até mesmo medidas de “reversão” (por exemplo, reinfusão de solução hipotônica e/ou desmopressina) em caso de sobrecorreção não intencional, especialmente em pacientes com maior risco para SDO (desnutrição, alcoolismo, hipopotassemia severa, doença hepática crônica)^2,6,7^.

Em suma, este trabalho original reproduz desfechos de outros estudos internacionais em população brasileira, contribuindo de forma significativa para a acalorada discussão das complicações do tratamento da hiponatremia grave, inclusive com a possibilidade futura de ajustes nas próprias *guidelines*.

## Data Availability

Nenhum dado novo foi gerado ou analisado neste estudo.
